# Validation of Songklanagarind Pediatric Triage Model in the Emergency Department; a Cross-Sectional Study

**DOI:** 10.22037/aaem.v9i1.1237

**Published:** 2021-05-20

**Authors:** Siriwimon Tantarattanapong, Nut Chonwanich, Wannipha Senuphai

**Affiliations:** 1 Department of Emergency Medicine, Songklanagarind Hospital, Faculty of Medicine, Prince of Songkla University, Hat Yai, Songkhla, Thailand.; 2 Nursing Department, Songklanagarind Hospital, Faculty of Medicine, Prince of Songkla University, Hat Yai, Songkhla, Thailand.

**Keywords:** Triage, pediatrics, reproducibility of results, emergency service, Hospital

## Abstract

**Introduction:**

An effective triage needs to consider many factors, such as good triage protocol, experienced triage nurses, and patient factors. This study aimed to evaluate the validity of Songklanagarind Pediatric Triage (SPT) for triage of pediatric patients in the emergency department (ED) and identify the factors associated with triage appropriateness.

**Methods:**

This study was done in two phases. In the first phase, a team of emergency physicians, a pediatric emergency physician, and a pediatric critical care physician developed SPT model by considering and combining Emergency Severity Index (ESI), Pediatric Assessment Triangle (PAT), Pediatric Canadian Triage and Acuity Scale (PaedCTAS), and Pediatric Septic Shock early warning signs protocol of the hospital as the core concept. In the second phase, a prospective observational study was conducted in the ED of Songklanagarind Hospital, which is a tertiary university hospital in southern Thailand, from September to October 2019 to evaluate the accuracy of the developed triage model.

**Results:**

A total of 520 pediatric patients met the inclusion criteria. The pediatric triage model had sensitivity and specificity values of 98.28% and 26.24%, respectively, and positive and negative predictive values of 27.67% and 98.15%, respectively, in prediction of death, hospitalization, and resource utilization. The rates of appropriate triage, over-triage, and under-triage were 68.8%, 28.5%, and 2.7%, respectively. Significant factors associated with appropriateness of triage were underlying disease of the respiratory system (OR = 4.16, 95%CI: 1.75‒9.23), fever (OR = 0.60, 95%CI: 0.41‒0.88), dyspnea (OR: 6.38, 95%CI: 2.51‒16.22), diarrhea (OR = 0.26, 95%CI: 0.09‒0.73), oxygen saturation <95% (OR = 3.18, 95%CI: 1.09‒9.27), accessory muscle use during breathing (OR = 3.67, 95%CI: 1.09‒12.41), and wheezing or rhonchi (OR = 6.96, 95%CI: 3.14‒15.43).

**Conclusion:**

SPT showed good correlation of hospital admission rates and resource utilization with pediatric triage level of urgency. However, further efforts are needed to decrease the rates of over- and under-triage.

## 1. Introduction

The volume of patients in the emergency department (ED) has recently increased, which has resulted in an imbalance between needs and resources (1). A qualified ED must have a standard 5-level triage system to prioritize patients according to severity and need for emergency management in the setting of limited resources (2). Patients with more urgent conditions need shorter waiting times to see the doctor. The safety of patients is the major issue. The factors that need to be considered for an effective triage are: a good triage protocol, experienced triage nurses, and patient factors (3). Special populations, especially pediatric patients, are challenging for triage. Special considerations, including age-specific vital signs and limited development of communication skills, influence the assessment of pediatric patients in the ED (3-5). 

Previous research that evaluated the validity of triage systems relied on two methods: 1) a comparison of the triage system with a reference standard developed by experts and 2) an association of the level of urgency and hospital admission or resource utilization (6). Under-triage increases the waiting time and increases morbidity and mortality. According to Hinson (2018), under-triage in moderate acuity had a critical outcome of 8.5%. Conversely, over-triage limits the time and resources for patients most in need (7). At the ED of Songklanagarind Hospital, which is a tertiary university hospital, the standard 5-level triage protocol was adapted from Emergency Severity Index (ESI) version 4.0. The results from a previous study at the ED showed an unexpected correlation between the admission rate and ESI level. The admission rates of ESI levels 1 to 5 were 57.1%, 21%, 42.2%, 1.4%, and 3.6%, respectively (8). 

Based on the results of the previous study, the Songklanagarind Pediatric Triage (SPT) was developed from the core concept of ESI version 4.0 and the initial assessment used by the Pediatric Assessment Triangle (PAT) (9). The Pediatric Canadian Triage and Acuity Scale (PaedCTAS) was used adjusted standard vital signs for each age group (10). In addition, the pediatric septic shock early warning sign protocol was used to develop SPT. 

The aim of this study was to evaluate the validity of SPT for triage of pediatric patients in the ED and identify the factors associated with triage appropriateness.

## 2. Methods

***2.1. Study design and setting***

This study was done in two phases. In the first phase (development phase) a team of emergency physicians, pediatric emergency physician, and the pediatric critical care physician developed the Songklanagarind Pediatric Triage (SPT) model by considering and combining ESI, PAT, PaedCTAS, and Pediatric Septic Shock early warning signs protocol of the hospital as the core concept. In the second phase (validation phase) a prospective observational study was conducted in the ED of Songklanagarind Hospital, which is a tertiary university hospital in southern Thailand, from September to October 2019 to evaluate the accuracy of the developed triage model. Ethics approval was obtained from the Institutional Ethics Committee Board of the Faculty of Medicine at Prince of Songkla University (Ethics code: REC.62-153-20-4.) 

***2.2. Development phase***

The ED of Songklanagarind Hospital uses the 5-level triage adapted from ESI version 4.0. Since the triage nurses were familiar with ESI, it was the core concept of SPT. In addition, PAT, PaedCTAS, and Pediatric Septic Shock early warning signs protocol of the hospital were used for further modifications and to set the high-risk situations and vital signs. 

**Figure 1:**
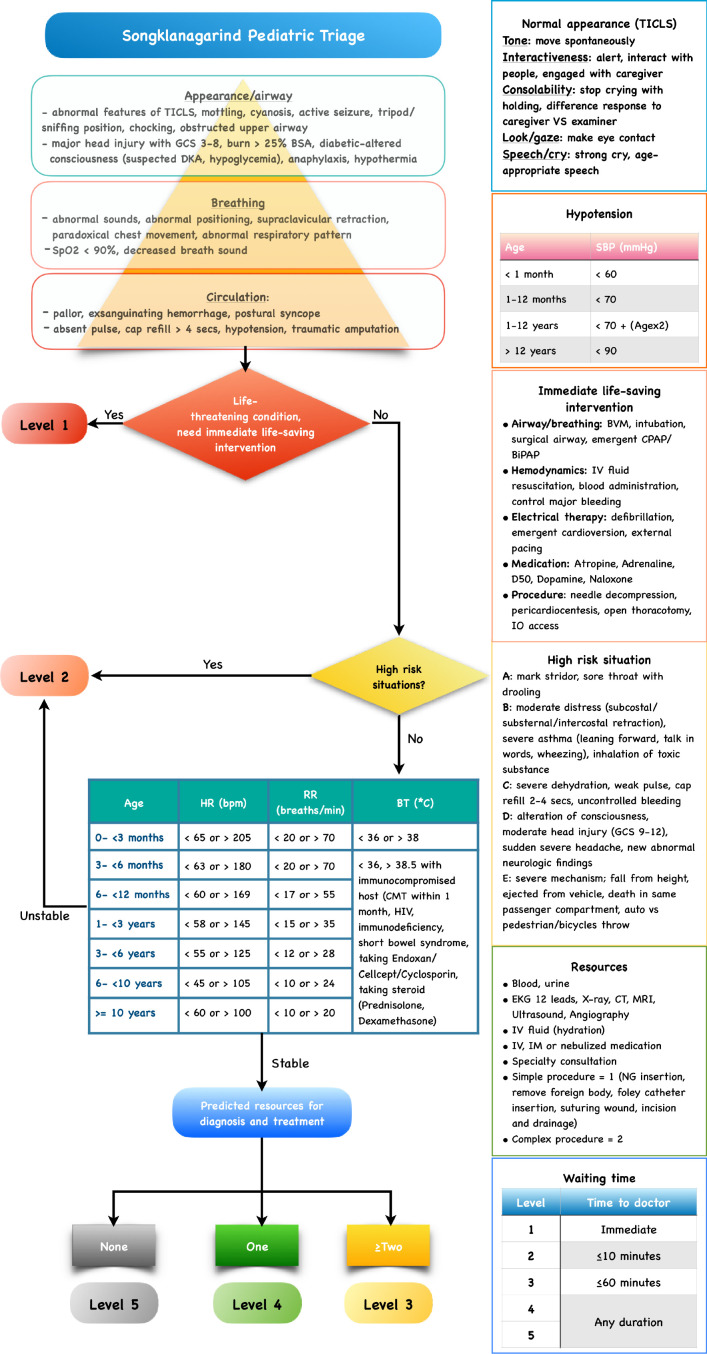
Songklanagarind Pediatric Triage

Based on the final developed model (Figure 1), pediatric patients arriving at the ED should be rapidly assessed by the triage nurses using PAT. An abnormal PAT or the need for life-saving intervention according to ESI version 4.0 led the patients to be categorized in pediatric triage level 1, who would immediately see the emergency physician (EP). Pediatric patients with high-risk situations or abnormal vital signs according to the PaedCTAS and the pediatric septic shock early warning signs protocol of the hospital were categorized in pediatric triage level 2 and would see the EP within 10 minutes. Pediatric patients with normal vital signs without high-risk characteristics were categorized according to the predicted number of resources for diagnosis and management. Pediatric patients with a prediction of ≥2 resources needed were categorized in triage level 3 and waited to see the EP within 60 minutes. Pediatric patients with a prediction of one resource needed were categorized in triage level 4, and those with a prediction of no resources needed were categorized in triage level 5. The waiting times in pediatric triage levels 4 and 5 were not guaranteed, but the triage nurses re-evaluated the patients to detect clinical deterioration during the waiting time.

SPT content validity was acceptable and the model was approved by the emergency physicians, pediatric emergency physician, and the pediatric critical care physician. The inter-rater reliability of SPT was evaluated using a scenario-based test, which yielded a Kappa value of 0.65.

***2.3. Validation phase***

***2.3.1. Participants***

Patients younger than 15 years who visited the ED were enrolled in this study. Patients excluded from the study were those scheduled for follow-up, those who were referred from other hospitals, and patients with incomplete medical records.

***2.3.2. Study protocol***

Before implementation of the pediatric triage, all triage nurses were trained and passed an examination regarding triage using SPT. The specific competency of the triage nurse consisted of experience working in the ED for more than 5 years and being well-trained in triage and advanced life support.

Pediatric patients were registered and assessed by the triage nurses. The nursing records were completed as much as possible with the basic information of the patients in addition to the initial assessment, chief complaint, signs, symptoms, and vital signs. The pediatric triage level was determined by the triage nurse using SPT (Figure 1) before seeing the physician. After completing the evaluation and treatment, the number of resources used including life-saving interventions, the final disposition, and diagnoses of the physicians were also recorded. 

Over-triage was defined as patients in pediatric triage levels 1, 2, or 3 who used <2 resources, or pediatric triage level 1 patients who were not admitted to the hospital (6). The definition of under-triage in this study consisted of patients in pediatric triage level 2 with an abnormal PAT or the need for an immediate life-saving intervention, patients in pediatric triage level 3 with abnormal vital signs or high-risk situations, and patients in pediatric triage level 4 or 5 who used ≥2 resources or were admitted to the hospital.

***2.3.3. Data gathering***

The data collected from the medical records included the patients’ baseline characteristics, arrival time, workday or weekend, mode of ED arrival, underlying diseases, chief complaint, initial vital signs, pediatric triage level, waiting time to see an EP, resources used, time and type of disposition, and final diagnosis. Also recorded were the factors that affected appropriate triage, such as overcrowding, which were the results from the National ED Overcrowding Study score (11), and the experience of the triage nurse.

***2.3.4. Outcome measurements***

The primary outcome was the validity of the pediatric triage to predict ED death, hospitalization, and resource utilization. The secondary outcome was evaluating the factors associated with inappropriate triage (over- or under-triage).

***2.3.5. Statistical analysis***

The data were entered into EpiData Manager **(**version 4.4.2.1) and the statistical analysis was conducted using R software **(**version 3.5.1). The sensitivity, specificity, positive predictive value (PPV), negative predictive value (NPV), and the rates of over- and under-triage were calculated. Continuous variables were analyzed and reported as median and interquartile range, while numeric variables were reported as percentage. All data had non-parametric frequency distributions. Continuous variables were compared using Kruskal-Wallis one-way analysis of variance (ANOVA). Numeric variables were compared using the chi-square test. Significant factors associated with appropriateness of triage were identified using odds ratio.

Screening performance characteristics of SPT model were calculated as follows: the sensitivity (ratio of admitted patients or deaths in the ED in pediatric triage levels 1‒3 and the total number of admitted patients or deaths in the ED), specificity (ratio of discharged patients in pediatric triage levels 4‒5 and the total number of discharged patients), PPV (ratio of admitted patients or deaths in the ED for pediatric triage levels 1‒3 and the number of patients in these levels), NPV (ratio of discharged patients of pediatric triage levels 4‒5 and the number of patients in these levels).

## 3. Results

***3.1. Baseline characteristic of studied cases***

A total of 546 pediatric patients visited the ED during the study period. Twenty-six patients were referred from other hospitals and were excluded. The number of patients who met the inclusion criteria was 520. The median age of the pediatric patients was 36 (12‒84) months and 53.7% were boys. Two hundred and eighty-eight (55.4%) patients visited the ED during the evening shifts and 284 (54.6%) patients presented during the workdays. The mode of arrival was most commonly walk-in, which was recorded for 498 (95.8%) patients. The percentage of non-traumatic chief complaints was 81.7%. The common chief complaints were fever (38.3%), dyspnea (12.5%), nausea and vomiting (7.9%), trauma related to the musculoskeletal system (7.3%), and abdominal pain (4.8%). No deaths were reported during the study.

***3.2. Screening performance characteristics of SPT***

The sensitivity, specificity, PPV, and NPV of SPT were calculated as 98.28% (95%CI: 93.91‒99.79), 26.24% (95%CI: 22.01‒30.82), 27.67% (95%CI: 26.43‒28.95), and 98.15% (95%CI: 93.00‒99.53), respectively.

***3.3. Appropriateness of triage using SPT***

The situation of resource consumption and final disposition in the different SPT levels are presented in Tables 1 and 2. The percentage of patients who were triaged appropriately was 68.7%, while 28.6% were over-triaged and 2.7% were under-triaged. Under-triage occurred in pediatric triage levels 2, 3, and 4 in 3, 1, and 10 patients, respectively. The definitive diagnoses in under-triaged patients were fracture (3 patients), anaphylaxis (2 patients), acute gastroenteritis (2 patients), septic shock (1 patient), vomiting with dehydration (1 patient), wheezing associated with respiratory infection (1 patient), dengue fever (1 patient), acute appendicitis (1 patient), acute bronchitis (1 patient), and urinary tract infection (1 patient). Over-triage occurred in pediatric triage level 2 (63 patients) and pediatric triage level 3 (86 patients). The common definitive diagnoses in over-triaged patients were acute gastroenteritis (22.6%), common cold (13.5%), dehydration (12.0%), acute febrile illness (11.3%), and limb injury (6.8%). 

Underlying disease of respiratory system, fever, dyspnea, diarrhea, oxygen saturation <95%, accessory muscle use, and wheezing or rhonchi in lung sounds were significantly associated with triage appropriateness (Tables 3 and 4). 

## 4. Discussion

Due to the high sensitivity and NPV of SPT, most of the high-urgency patients were rapidly detected and most patients with a low level of urgency truly had non-urgent conditions. These results implied that SPT is a good screening tool for most urgent cases and indicated a high probability of detecting actual non-urgent patients in pediatric triage levels 4 and 5. Low specificity indicates that few patients with non-urgent conditions were correctly detected and is represented by the high rate of over-triage.

Special considerations that affect triage in pediatric patients are age-specific vital signs and the limited development of communication. In a study by Cooper et al. (12), 1130 triage decisions based on history, visual cues, limited physical examination, and incomplete vital signs should have been revised. Seventy percent of these changes were to a more urgent level and 20.7% of the patients were under 15 years old. Categorizing the patient in a less urgent level due to incomplete assessment resulted in longer waiting times, which affected patient safety (13). This study showed that oxygen saturation was associated with appropriateness of the triage decision and this measurement was missed in only 4.6%.

Chief complaints such as fever and diarrhea need to be carefully evaluated, because these conditions affect the vital signs and result in tachycardia or tachypnea, which increase the rate of over-triage. However, tachycardia in a pediatric patient can be caused by either serious or non-serious conditions. Tachycardia is an early sign of shock but non-specific conditions such as crying and fever may also be the cause. If the triage nurse strictly follows the protocol, the rate of over-triage will increase. This is an acceptable issue for patient safety.

The rate of hospital admission in each level from 1 to 5 of this study and the previous study in Songklanagarind Hospital (8) were 100%, 31.6%, 16.8%, 2%, and 0% and 57.1%, 21%, 42.2%, 1.4%, and 3.6%, respectively. Therefore, as a triage tool, SPT showed greater validity. The percentage of patients in each level from 1 to 5 who needed more than one resource decreased and resource consumption increased from level 1 to 5. The data also presented correlated outcomes of hospital admission and resource utilization across the five levels of urgency, which were comparable to a previous systematic review of standard triage tools by de Magalhães-Barbosa et al. (6).

Even though the ED uses the same standard international triage tool, the validity of a triage tool can vary. Based on institutional studies, development of the SPT, which is compatible with specific patient characteristics and the local health system, permitted high validity and appropriate resource utilization. 

**Table 1:** Numbers of resources consumed in different levels of the Songklanagarind Pediatric Triage

**Triage level**	**≥** **2 resources**	**1 resource**	**0 resource**
1 (n = 10)	10 (100)	0	0
2 (n = 247)	156 (63.2)	73 (29.6)	18 (7.3)
3 (n = 155)	67 (43.2)	66 (42.6)	22 (14.2)
4 (n = 100)	10 (10.0)	56 (56.0)	34 (34.0)
5 (n = 8)	0 (0.0)	2 (25.0)	6 (75.0)

Data are presented as number (%).

**Table 2:** Songklanagarind Pediatric Triage level and disposition

**Triage level**	**Hospitalization**				**Discharge**
	**Total **	**ICU**	**Ward**	**ED OR **	
1 (n = 10)	10 (100)	4 (40.0)	6 (60.0)	0 (0.0)	0 (0.0)
2 (n = 247)	78 (31.6)	3 (1.2)	75 (30.4)	0 (0.0)	169 (68.4)
3 (n = 155)	26 (16.8)	0 (0.0)	24 (15.5)	2 (1.3)	129 (83.2)
4 (n = 100)	2 (2.0)	0 (0.0)	2 (2.0)	0 (0.0)	98 (98.0)
5 (n = 8)	0 (0.0)	0 (0.0)	0 (0.0)	0 (0.0)	8 (100.0)

Data are presented as number (%). ICU: intensive care unit; ED: emergency department; OR, operation room.

**Table 3:** Associated factors of appropriate (n = 357) and inappropriate (n = 163) triage

**Variable**	**Appropriate **	**Inappropriate**	**P**
**Male**	189 (52.9)	90 (55.2)	0.698
**Age, median ** **(** **IQR** **)** **, months**	36 (12, 84)	36 (12, 84)	0.536
**Thai ethnicity**	356 (99.7)	162 (99.4)	0.529
**Work shift**			
Morning shift	85 (23.8)	43 (26.4)	0.739
Evening shift	198 (55.5)	90 (55.2)	
Night shift	74 (20.7)	30 (18.4)	
**Workday**	196 (54.9)	88 (54.0)	0.921
**ED arrival by self-transport**	340 (95.2)	158 (96.9)	0.742
**Underlying disease**	93 (26.1)	29 (17.8)	**0** **.** **051**
**Trauma cause**	70 (19.6)	25 (15.3)	0.295
**Non** **-** **trauma cause**	287 (80.4)	138 (84.7)	
**Chief complaint**			
Fever	123 (34.5)	76 (46.6)	**0** **.** **011**
Dyspnea	60 (16.8)	5 (3.1)	**<0** **.** **001**
Nausea and vomiting	23 (6.4)	18 (11.0)	0.103
Abdominal pain	14 (3.9)	11 (6.7)	0.239
Diarrhea	6 (1.7)	10 (6.1)	**0** **.** **014**
Seizure	9 (2.5)	1 (0.6)	0.183
Alteration of consciousness	2 (0.6)	0	1.000
**Abnormal ** **primary assessment triangle** ** (** **PAT** **)**			
Airway problem	6 (1.7)	0	NA
Abnormal breathing	4 (1.1)	0	
Abnormal circulation	0	0	
**Vital signs in triage area**			
Body temperature	345 (96.6)	159 (97.5)	0.778
Systolic blood pressure	344 (96.4)	158 (96.9)	0.941
Pulse rate	353 (98.9)	163 (100)	0.314
Respiratory rate	339 (95.0)	155 (95.1)	1.000
Oxygen saturation	339 (95.0)	157 (96.3)	0.645
SaO_2_ <95%	26 (7.7)	4 (2.5)	0.043
**Crying#**	46 (12.9)	20 (12.3)	0.526
**Accessory muscle use**	23 (6.4)	3 (1.8)	**0** **.** **044**
**Wheezing, rhonchi **	85 (23.8)	7 (4.3)	**<0** **.** **001**
**Full pulse**	356 (99.7)	163 (100)	1.000
**Full consciousness **	348 (97.5)	159 (97.5)	1.000
**Nurses’ experience ** **(** **>10 years** **)**	220 (61.6)	108 (66.3)	0.359
**Overcrowding** *	211 (59.1)	96 (58.9)	1.000

*: Numbers of patients visited during overcrowding periods.

#: Crying during vital signs measurement.

Data are presented as number (%) unless otherwise indicated.

Morning shift 8:00 AM–4:00 PM; Evening shift 4.00 PM–0:00 AM; Night shift 0:00 AM–8:00 AM.

IQR: interquartile range; ED: emergency department.

**Table 4:** Odds ratios of factors associated with appropriateness of triage

**Variable**	**Odds ratio**	**95** **%** **CI**
Underlying disease of respiratory system	4.16	(1.75‒9.23)
Fever	0.60	(0.41‒0.88)
Dyspnea	6.38	(2.51‒16.22)
Diarrhea	0.26	(0.09‒0.73)
SaO_2_ <95%	3.18	(1.09‒9.27)
Accessory muscle use	3.67	(1.09‒12.41)
Wheezing or rhonchi	6.96	(3.14‒15.43)

CI: confidence interval; SaO_2_: Oxygen saturation.

## 5. Limitations

A limitation of this study was that the admission rates of modified ESI and pediatric triage were compared in different populations. Another limitation was the lack of information on whether the patients were admitted to other healthcare facilities after leaving the ED.

## 6. Conclusion

SPT showed good correlation of hospital admission rates and resource utilization with pediatric triage level of urgency. However, further efforts are needed to decrease the rates of over- and under-triage. Further discussion between the experts and multidisciplinary team is needed for quality improvement.

## 7. Declarations

### 7.1. Acknowledgments

The authors thank Kingkarn Waiyanak for searching for articles and retrieval. The authors thank Teeranai Sakulchit MD, Pediatric Emergency Physician, Department of Emergency Medicine and Kantara Saelim MD, Division of Pulmonary and Critical Care, Department of Pediatrics for reviewing the triage protocol. The authors also thank Glenn K. Shingledecker for his help in editing the English of the manuscript.

### 7.2. Author contributions

Nut Chonwanich performed the literature research, study design, data collection, data analysis, data interpretation, and writing the manuscript. Wannipha Senuphai did data collection, data analysis, and data interpretation. Siriwimon Tantarattanapong did the study design, data analysis, data interpretation, critical revision, and writing the manuscript. The authors contributed to data analysis, drafting, and the critical revisions of the paper and agree to be accountable for all aspects of the work.

### 7.3. Conflict of interest

The authors declare they have no conflict of interest.

### 7.4. Funding and supports

The Faculty of Medicine, Prince of Songkla University funded this research.
